# Supplementary material for: Zinc oxide nanoparticles modulate the gene expression of ZnT1 and ZIP8 to manipulate zinc homeostasis and stress-induced cytotoxicity in human neuroblastoma SH-SY5Y cells
Source: PLoS One. 2020 Sep 11;15(9):e0232729. doi: 10.1371/journal.pone.0232729 (PMC7485861; doi:10.1371/journal.pone.0232729)
Supplement: S1 File — (DOCX) [file pone.0232729.s001.docx]

**Supporting Information**

**Zinc oxide nanoparticles modulate the gene expression of ZnT_1_ and ZIP_8_ to manipulate zinc homeostasis and stress-induced cytotoxicity in human neuroblastoma SH-SY5Y cells**

Chien-Yuan Pan^1^, Fang-Yu Lin^2^, Lung-Sen Kao^3,4^, Chien-Chang Huang^4^, Pei-Shan Liu^2*^

^1^Department of Life Science and Institute of Zoology, National Taiwan University, Taipei, Taiwan

^2^Department of Microbiology, Soochow University, Taipei, Taiwan

^3^Brain Research Center, National Yang-Ming University, Taipei, Taiwan

^4^Department of Life Sciences and Institute of Genome Sciences, National Yang-Ming University, Taipei, Taiwan

*Correspondence: Dr. Pei-Shan Liu, Department of Microbiology, Soochow University,

No. 70, LinXi Road, Shihlin District, Taipei 111, Taiwan, R. O. C. Tel: 886-2-28819471 ext. 6857; Fax: 886-2-28831193 Email: psliu@ scu.edu.tw.

**Table S1. Primer sequences**

| gene name | F/R | 5'-->3' |
| --- | --- | --- |
| ZnT1 | F/R | GTGTAGTTCCGTGTGAACTTGCC/ AAATGCTAACTGCTGGGGTCTTTTC |
| ZnT2 | F/R | GGGGCTTGTGTGTGAAGATT/ GCCAACTGGCTCTTGTTCTC |
| ZnT3 | F/R | TGCAGGTCGAGCAGTATCAG/ GTGGTAGGAGGGAGAGAGG |
| ZnT4 | F/R | TCTGGGTGTGAACGTAACCA/ GATGGGGTCAGCAATCTTGT |
| ZnT5 | F/R | GCCCTGATGAGTAGGTGGAA/ TCAGCCCTCCAACTGAGACT |
| ZnT6 | F/R | GCCGTGTCCTGAGTTATGGT/ GGTGTTGCCTCTGAATTGGT |
| ZnT7 | F/R | ACCTCCCTGCCTTTAGTGGT/ TGCACTTGGGCAAATCATAA |
| ZnT8 | F/R | AGCCTGCACATCTGGTCTCT/ GGGAAACTGACGGTGTGACT |
| ZnT9 | F/R | GATTTTGATGGGCGAGTTGT/ CCCAGGTGATTCATTCCATC |
| ZnT10 | F/R | AGCCTGACTGTCCTCATGGT/ GTGCAGGGTGGCAATAATCT |
| ZIP1 | F/R | CCCAAGGAACAAGAGATGG/ CTGAAATGGGCTAGGACCAA |
| ZIP2 | F/R | ATCATCTCCCTGGGCTTCTT/ CCACAGCTAGCCCTTCAAAC |
| ZIP3 | F/R | CTGTGAGGGAAAAGCTCCAG/ GAAGGTCTCCAGGTCGATGA |
| ZIP4 | F/R | GTAGTTGGGGAAGCAGGACA/ TGTGGGCAGAGACAAGTGAG |
| ZIP5 | F/R | GGGTGACCTGGAAGAGTCAA/ CAGCAGAGCAAACTGACGAG |
| ZIP6 | F/R | AGAGCCCTCCCACTTTGATT/ GCCGAGTGTATCGTGGAAAT |
| ZIP7 | F/R | GGCTGGCAGTCTTACAGAGG / CAACTGGTGGGAGAAAGGAA |
| ZIP8 | F/R | TCCTGCACCTTGTCTCTCCT / GCCCAACATAGCAGGAACAT |
| ZIP9 | F/R | TGTCTGGTTTGGTTTGGACA / TAAGAGGGCCCTGAAAGGTT |
| ZIP10 | F/R | CCTTTGCCCTGCATTGTTAT/ AGATCACGCCTAGCAAGGAA |
| ZIP11 | F/R | CAGGGGTAGCAATGACCTGT/ CAGGAGATTCACCCTTGGAA |
| ZIP12 | F/R | ACCCTCCGCCTATCAGAACT/ TGAGTGAGAGGCCCTTCTGT |
| ZIP13 | F/R | ATGGTGCTGTTCTCGCTCTT / CATACATCCACACGCACCTC |
| ZIP14 | F/R | TATGGAGAACCACCCCTGAG/ CACGTGCTGGGTGACATTAC |
| MT-1A | F/R | CGTGCGCCTTATAGCCTCTC/ TCTCTGATGCCCCTTTGCAG |
| MT3 | F/R | AAGTGTGCCAAGGACTGTGT/ CACCTGGCACTATCTCCACG |
| MT-3 | F/R | CCGGAATTCCACCGTTGCTCCAGATTCAC/ CCCAAGCTTGGTGTACGGCAAGACTCTGA |
| P53 | F/R | AGCCAAGTCTGTGACTTGCA/ AACCTCCGTCATGTGCTGT |
| Bax | F/R | AACTGGTGCTCAAGGCCCTG/ GGGTGAGGAGGCTTGAGGAG |
| Bcl-2 | F/R | TTTGAGTTCGGTGGGGTCATG/ TCACTTGTGGCCCAGATAGGC |
| β-actin-1 | F/R | GGCACTCTTCCAGCCTTCCT/ TGCGCTCAGGAGGAGCAATG |
| β-actin-2 | F/R | CCTTCCTGGGCATGGAGTCCTG / GGAGCAATGATCTTGATCTTC |

**Table S2 shRNA sequences against ZnT_1_ and ZIP_8_**

| five shRNA target sequences for human ZnT1 | |  |  |
| --- | --- | --- | --- |
| shRNA-H1 | 5′-GCTACTACCATTCAGCCTGAA-3′ | |  |
| shRNA-H2 | 5′- GTTCAGTGATTGTAGTAGTAA-3′ | |  |
| shRNA-H3 | 5′-CCTTCTGGA AAGGATGCAGAA3′ | |  |
| shRNA-H4, | 5′- CCTGCAAAGCATTTGTAGAAA-3′ | |  |
| shRNA-H5 | 5′- GAAGTACAAGTGAATGGAAAT-3′ | | |
| Four shRNA target sequences for human ZIP8 | | | |
| shRNA-H6 | 5′- GCTGCACTTCAACCAGTGTTT-3′ | | |
| shRNA-H7 | 5′- CATGATTC AGAATGCTGGAAT-3′ | | |
| shRNA-H8 | 5′- GCCAAGTTCATGTACCTGTTT-3′ | | |
| shRNA-H9 | 5′- CCTGTCAGTGACGATTATTAA-3′ | | |

**Figure S1**

**Figure S1** The statistical symbols at each time point when compared to the control group. The data shown in Fig. 1B were replotted as a bar graph to include the statistical symbols.

**Figure S2**

**Figure S2** The expressions of ZnT and ZIP isoforms in SH-SY5Y cells. Cells were harvested for RT-PCR with specific primers against different ZnT_1-10_ and ZIP_1-14_ isoforms. M: 100 bp markers; a: the product of primers against β-actin.

**Figure S3**

**Figure S3** Western blot images of ZnT_1_ and ZIP_8_ from cells transfected with shRNA. Cells were transfected with shRNA against ZnT_1_ (H1-H5), ZIP_8_ (H6-9) or negative control (ctl) and then harvested for Western blot with polyclonal antibodies against ZnT_1_ (A) and ZIP_8_ (B). The red rectangle indicates the regions shown in Fig. 2 C & D.

**Figure S4**

**Figure S4** The expression of *Bax*, *Bcl-2*, and *Actin* shown in Fig. 3B. Shown on the left were the original images of the agarose gel analysis for the PCR products of *Bax*, *Bcl-2*, and *β-actin* (From top to bottom, respectively). These bands were cropped and used in Fig. 3B. To highlight the 100 bp markers, the brightness and contrast of these images were adjusted and shown on the right.

**Figure S5**

**Figure S5** ZnO-NP treatment reduces ROS production. Cells were treated with ZnO-NP (0, 0.081 and 0.814 μg/ml) for 24 hr, then loaded with H_2_DCFDA to monitor the amount of ROS accumulation for another 120 min.

**Figure S6**

**Figure S4** ZnO-NP suppressed H_2_O_2_-induced cytotoxicity and ROS production in SH-SY5Y cells. A. Cell viability. Cells were pretreated with ZnO-NP (0.081 μg/ml) for 20 hr and then incubated with H_2_O_2_ for 4 hr. The survival rate was determined by MTT assay from 5 batches of cells. B. ROS production. Cells were pretreated with ZnO-NP (0.081 μg/ml) for 23 hr and then incubated with H_2_O_2_ for another 1 hr. The intracellular ROS was determined by the fluorescence intensity of loaded H_2_DCFDA. Data presented were Mean ± SEM (n = 12) and the significance were analyzed by Student’s *t*-test; **: *p* < 0.01 when compared to the control group without 6-OHDA treatment or as indicated.

**Figure S7**

**Figure S7** The original agarose gel analysis of the expression of *p53* and *β-actin* in SH-SY5Y cells. The red rectangles indicate the regions shown in Fig. 4C.
